# METCAM/MUC18 is a novel tumor and metastasis suppressor for the human ovarian cancer SKOV3 cells

**DOI:** 10.1186/s12885-016-2181-9

**Published:** 2016-02-22

**Authors:** Guang-Jer Wu, Guo-fang Zeng

**Affiliations:** Department of Microbiology and Immunology, Emory University School of Medicine, Atlanta, GA 30322 USA; Department of Bioscience Technology, Chung Yuan Christian University, Chung Li, 32023 Taiwan; Center for Biomedical Technology, Chung Yuan Christian University, Chung Li, 32023 Taiwan; Present Address: Department of Hepatobiliary Surgery, Institute of Plastic Surgery, and Laboratory of Regenerative Medicine, Affiliated Hospital of Guangdong Medical College, Zhanjiang, 542001 China

**Keywords:** Human METCAM/MUC18 expression, Ovarian cancer SKOV3 cells, *SC & IP* injections, Tumorigenesis and progression, Athymic nude mice

## Abstract

**Background:**

Increased expression of METCAM/MUC18, a trans-membrane cell adhesion molecule in the Ig-like gene superfamily, has been associated with the malignant progression of epithelial ovarian carcinomas. To investigate if this is a fortuitous correlation or if METCAM/MUC18 actually plays a role in the progression of the cancer, we tested effects of enforced expression of METCAM/MUC18 on in vitro behaviors, in vivo tumorigenesis, and in vivo malignant progression of human ovarian cancer SK-OV-3 cells, which minimally expressed this protein.

**Methods:**

For in vitro and in vivo tests, we transfected human METCAM/MUC18 cDNA gene into SK-OV-3 cells in a mammalian expression vector pcDNA3.1+ and obtained G418-resistant (G418^R^) clones, which expressed various levels of human METCAM/MUC18. To mimic physiological situations, we used pooled METCAM/MUC18-expressing and control (vector) clones for testing effects of human METCAM/MUC18 over-expression on in vitro motility and invasiveness, and on in vivo tumor formation and metastasis in female athymic nude mice. Effects of METCAM/MUC18 on the expression of various downstream key factors related to tumorigenesis were also evaluated by Western blot analyses.

**Results:**

The over-expression of METCAM/MUC18 inhibited in vitro motility and invasiveness of SK-OV-3 cells. SK-OV-3 cells of the control (vector) clone (3D), which did not express human METCAM/MUC18, supported the formation of a solid tumor after *SC* injection of the cells at dorsal or ventral sites and also formation of solid tumor and ascites after *IP* injection in the intraperitoneal cavity of nude mice. In contrast, SK-OV-3 cells from the METCAM/MUC18-expressing clone (2D), which expressed a high level of METCAM/MUC18, did not support the formation of a solid tumor at *SC* sites, or formation of ascites in the intraperitoneal cavity of nude mice. Expression levels of downstream key factors, which may affect tumor proliferation and angiogenesis, were reduced in tumors induced by the METCAM/MUC18-expressing clone (2D).

**Conclusions:**

We conclude that increased human METCAM/MUC18 expression in ovarian cancer SK-OV-3 cells suppressed tumorigenesis and ascites formation in nude mice, suggesting that human METCAM/MUC18 plays a suppressor role in the progression of ovarian cancer, perhaps by reducing proliferation and angiogenesis.

## Background

Epithelial ovarian cancer (EOC) is the fifth leading cause of female cancers in USA with a high fatality rate (about 65 %) [1]. The high lethality of the cancer is because the early stage of the disease is mostly asymptomatic and therefore remains undiagnosed until the cancer has already disseminated throughout the peritoneal cavity [2]. The early stage disease can be treated successfully, however, effective therapy for the advanced-stage disease is lacking because of the strong chemo-resistance of recurrent ovarian cancer [2]. The major challenges for combating ovarian cancer are: (a) the ovarian cancer is histologically and molecularly heterogeneous with at least four major subtypes [3, 4], (b) there is a lack of reliable specific diagnostic markers for an effective early diagnosis of each subtype, though molecular signatures of the major subtypes are available [5], and (c) very little is known of how ovarian tumor emerges and how it progresses to malignancy ([6] for a review).

In general, tumorigenesis is a complex process involving changes of several biological characteristics [7], including the aberrant expression of cell adhesion molecules [8]. Tumor progression is induced by a complex cross-talk between tumor cells and stromal cells in the surrounding tissues [8]. These interactions are, at least in part, mediated by cell adhesion molecules (CAMs), which govern the social behaviors of cells by affecting the adhesion status of cells and cross-talk and modulating intracellular signal transduction pathways [8]. Thus the altered expression of CAMs can change motility and invasiveness, affect survival and growth of tumor cells, and alter angiogenesis [8]. As such, CAMs may promote or suppress the metastatic potential of tumor cells [9]. Aberrant expression of various CAMs, such as mucins [10], integrins [11], CD44 [12], L1CAM [13], E-cadherin [14], claudin-3 [15], EpCAM [16], and METCAM/MUC18 [17, 18], has been associated with the malignant progression of ovarian cancer.

We have been focusing our studies on the possible role of METCAM/MUC18 in the progression of several epithelial tumors [19]. Human METCAM/MUC18 (or MCAM, Mel-CAM, S-endo1, or CD146), an integral membrane cell adhesion molecule (CAM) in the Ig-like gene superfamily, has an N-terminal extra-cellular domain of 558 amino acids, a transmembrane domain, and a short intra-cellular cytoplasmic domain (64 amino acids) at the C-terminus [19, 20]. The extra-cellular domain of the protein comprises a signal peptide sequence and five immunoglobulin-like domains and one X domain [19, 20]. The cytoplasmic domain contains five consensus sequences potentially to be phosphorylated by PKA, PKC, and CK2 [19, 20]. Thus human METCAM/MUC18 is capable of performing typical functions of CAMs, such as governing the social behaviors by affecting the adhesion status of cells and modulating cell signaling. Therefore, an altered expression of METCAM/MUC18 may affect motility and invasiveness of many tumor cells in vitro and tumorigenesis and metastasis in vivo [19].

Human METCAM/MUC18 is only expressed in several normal tissues, such as hair follicular cells, smooth muscle cells, endothelial cells, cerebellum, normal mammary epithelial cells, basal cells of the lung, activated T cells, and intermediate trophoblasts [19, 21]. Human METCAM/MUC18 is also expressed in several epithelial tumors, such as melanoma, prostate cancer, osteosarcoma, breast carcinoma, and intermediate trophoblast tumors [19, 21]. Over-expression of METCAM/MUC18 promotes the tumorigenesis of prostate cancer [22] and breast carcinoma [23, 24], but it has a minimal effect on the tumorigenesis of melanoma [25]. Over-expression of METCAM/MUC18 also initiates the metastasis of prostate cancer [26] and promotes the metastasis of melanoma [25] and breast carcinoma [27].

On the contrary, the possibility that the over-expression of METCAM/MUC18 might play a tumor suppressor role was first suggested by Shih et al. [28], who found that METCAM/MUC18 expression suppressed tumorigenesis of a breast cancer cell line MCF-7 in SCID mice. However, this notion was contradicted by recently published evidence, which supported the positive role of METCAM/MUC18 in the progression of breast cancer cells [23, 24, 27], similar to its role in the progression of melanoma and prostate cancer cells.

The role of METCAM/MUC18 in the progression of ovarian cancer has not been well studied, except that the METCAM/MUC18 expression has been recently reported to correlate with the progression of ovarian cancer [17, 18], and perhaps affects the behaviors of ovarian cancer cells [29]. To directly test the role of METCAM/MUC18 in the progression of epithelial ovarian cancer, we first chose to use SK-OV-3 cells for testing the effect of over-expression of METCAM/MUC18 on in vitro motility and invasiveness, in vivo tumor formation in nude mice after subcutaneous (*SC*) injection, and in vivo progression in nude mice after intraperitoneal (*IP*) injection. We found that the over-expression of METCAM/MUC18 inhibited in vitro motility and invasiveness and suppressed in vivo tumorigenesis and the malignant progression of the human ovarian cancer cell line SK-OV-3. We conclude that METCAM/MUC18 is a novel tumor and metastasis suppressor for the progression of human ovarian cancer cells.

## Methods

### Cell lines and culture

SK-Mel-28, a human melanoma cell line from ATCC, which was maintained in EMEM supplemented with 1 mM Na.pyruvate, extra nonessential amino acids and vitamins, and 10 % fetal bovine serum (FBS), was used as a positive control (100 %) for the expression of human METCAM/MUC18. LNCaP, a human prostate cancer cell line from ATCC, which was maintained in modified RPMI 1640 medium supplemented with 25 mM HEPES, 1 mM Na.pyruvate, 1 mM glutamine, and 10 % FBS, was used as a negative control (0 %) for the expression of human METCAM/MUC18. Human ovarian cancer cell lines, CAOV3, SK-OV-3, and NIHOVCAR3, were from ATCC. CAOV3, which was established from human primary ovarian adenocarcinoma, was maintained in DMEM (4.5 g/L of glucose) and 10 % FBS. SK-OV-3, which was established from malignant ascites of human ovarian adenocarcinoma, was maintained in McCoy’s 5A medium with 10 % FBS. NIHOVCAR3, which was established from malignant ascites of human ovarian progressive adenocarcinoma, was maintained in modified RPMI medium-4.5 g/L glucose-1 mM Na.pyruvate, 10 μg/ml insulin, and 20 % FBS. IOSE from Dr. Nelly Auesperg, Vancouver, Canada, which was a normal human ovarian surface epithelial cell line immortalized by the SV40 virus large T antigen [30], was maintained in M199/MCDB105 (1:1) medium with 15 % FBS and 50 μg/ml of gentamicin. BG-1 from Drs. Erin Dickerson and Nathan Bowen at Georgia Institute of Technology, Atlanta, GA, which was established from poorly differentiated human primary ovarian adenocarcinoma [31], was maintained in DMEM/F12 with 10 % FBS. HEY from Dr. Gordon Mills at M.D. Anderson Cancer Center, Houston, TX, which was established from a mouse xenograft of human primary ovarian adenocarcinoma [32], was maintained in a modified RPMI 1640 medium supplemented with 25 mM HEPES, 1 mM Na.pyruvate, 1 mM glutamine, 4.5 g/L glucose and 10 % FBS. All the SK-OV-3 clones were maintained in the McCoy’s 5A medium with 10 % FBS plus 0.5 mg/ml of G418. All media were from Invitrogen/Life Technology/GIBCO/BRL. FBS was from Cellgro/MediaTech. All the cell lines and SK-OV-3 clones were maintained in a humidified 37 ° C incubator with 5 % CO_2_.

### Lipofection of SK-OV-3 cells and selection for human METCAM/MUC18-expressing clones

1 × 10^6^ of SKOV3 cells per well were seeded (about 60 % confluence) in 6-well plates 1 day before lipofection. Lipofection was carried out with a mixture in 2 ml of Opti-MEM containing 12 μg of DEMRIE-C, or 6 μg of FuGene HD (Cat.no.04-709-691-001, Roche), and 2 μg each of the plasmid pcDNA3.1+ with or without the human METCAM/MUC18 cDNA gene for 6 h at 37 C. At the end of lipofection, 0.2 ml FBS was added to make the final serum concentration to 10 %. After cultured for two more days, the transfected cells were split into two plates containing the growth medium plus 0.5 mg/ml of G418 (active component 71.3 %). G418-resistant (G418^R^)-clones emerged in 2 weeks. Twelve clones from each lipofection were picked, transferred and expanded sequentially from 24-well to 12-well and 6-well culture plates. Cell lysate of each clone grown in each well of 6-well plates was made by addition of 100 μl of Western blot lysis buffer [22–24] and processed for Western blot analysis [22–24]. Liquid-nitrogen-frozen stocks of the METCAM/MUC18-expressing clones (METCAM/MUC18 clones) and the control (vector) clones were made from duplicated 6-well plates. The single METCAM/MUC18-expressing clones were designated as METCAM/MUC18 clone 2D-1 to 2D-12 (or abbreviated as METCAM clone 2D-1 to 2D-12). After single colonies were picked, the remaining colonies in the plates were treated with trypsin and pooled together, and seeded to duplicate T-25 flasks. After growth, cells from the pooled clones in one flask were frozen and designated either as METCAM/MUC18 clone 2D (or abbreviated as METCAM clone 2D) or control (vector) clone 3D, and those in another flask were made Western blot lysate, designated as cell lysate of METCAM clone 2D or control (vector) clone 3D.

### Cell motility assay

The in vitro cell motility assay was carried out [23–26]. 2 × 10^5^ cells of the METCAM clone 2D or the control (vector) clone 3D of SK-OV-3 cells in 0.4 ml of growth medium containing 0.1 %-BSA were seeded to each of the top insert with 8.0 μm pore size of the polycarbonate membrane (Fisher #08-771-12 or Falcon 35-3182) that fits into the bottom wells of a companion 12-well plate of the Boyden type Transwell system (Fisher #08-771-22 or Falcon 35-3503). Each bottom-well was added 1.1 ml of regular growth medium containing 10 % FBS. After 6 h, cells migrating to the bottom wells were treated with trypsin, concentrated by centrifugation, and counted with a hemocytometer [23–26]. The mean value and the standard deviation of three measurements of cell numbers migrated to bottom wells were calculated and presented.

### Cell invasiveness assay

The in vitro cell invasiveness assay was carried out [23–26]. All procedures were similar to the cell motility assay except each top well (with a pore size of 12 μm) was coated with 150 μg of diluted Matrigel (growth factors-reduced and phenol-red free grade, BD Biosciences Cat # 354237 or Collaborative Research Cat. #40234C). After 6 h, cells migrating to the bottom wells were determined. The mean value and the standard deviation of three measurements of cell numbers migrated to bottom wells were calculated and presented.

### Determination of tumorigenesis of SK-OV-3 clones/cells at the subcutaneous (*SC*) sites of athymic nude mice

All animal studies complying with the Institutional, national and international guidelines were approved by the Emory University’s animal ethics committee, Institutional Animal Care and Use Committee (IACUC), with an approval ID of 275-2008 (from 2/16/2009 to 2/16/2011). Emory’s Animal Welfare Assurance Number is A3180-01. Ten 33 days-old female athymic nude mice from Harlan Sprague Dawley Inc. (Indianapolis, Indiana, USA) were used for *SC* injection of cells from each clone. A single cell suspension was made from monolayer cultures of SK-OV-3 clones/cells after trypsin treatment, washed, re-suspended in PBS (5 × 10^6^ cells/ml), cooled in ice, centrifuged, re-suspended in 0.05 ml of cold McCoy’s 5A medium without FBS, and mixed with an equal volume of Matrigel (16 mg/ml, Cultrex, Trevigen) to make a final concentration of 5 × 10^7^ cells per ml and Matrigel at 8 mg/ml [22–25]. 5 × 10^6^ cells of the METCAM clone 2D (p24) and the control (vector) clone 3D (p24) of SK-OV-3 cells in 0.1 ml were subcutaneously injected with a gauge #28G1/2 needle into the right dorsal flank or the right ventral side. After injection, the size of tumor was weekly measured with a caliper till 40 days. Tumor volumes were calculated by using the formula V = π/6 (d1 × d2)^3/2^ (mm)^3^ [22–25]. At the endpoint, mice were euthanatized, tumor from each mouse was excised, weighed, and a portion was made cell lysate for Western blot analysis. The rest of the tumor was fixed in phosphate-buffered 10 % formaldehyde (Fisher), paraffinized, and sectioned for histology and immunohistochemistry staining.

### Determination of tumorigenesis and progression of SK-OV-3 clones/cells in the intra-peritoneal cavity of female athymic nude mice

All animal studies complying with the Institutional, national and international guidelines and were approved by the Emory University’s animal ethics committee, Institutional Animal Care and Use Committee (IACUC), with an approval ID of 275-2008 (from 2/16/2009 to 2/16/2011). Emory’s Animal Welfare Assurance Number is A3180-01. Five 34 days-old female athymic nude mice from Harlan Sprague Dawley Inc. were used for *IP* injection of cells from each clone [22–26]. A single cell suspension was made from monolayer cultures of SK-OV-3 clones/cells after trypsin treatment, washed, re-suspended in PBS (3 × 10^7^ cells /ml), cooled in ice, centrifuged, and re-suspended in 2 ml of cold PBS, and mixed with 1 ml of cold Matrigel (16 mg/ml, Cultrex, Trevigen) to make a final concentration of 1 × 10^7^ cells per ml and Matrigel at 5.55 mg/ml [22–25]. 5 × 10^6^ cells of the METCAM clone 2D (p19) and the control (vector) clone 3D (p19) of SK-OV-3 cells in 0.5 ml containing Matrigel were injected into intra-peritoneal cavity. The formation of solid tumors and ascites in the abdomen of each mouse was weekly monitored till the end of the experiments (10 weeks). After euthanasia, ascites were carefully withdrawn from abdominal cavities with pipets and total volumes of ascites were recorded. Ascites were centrifuged at 700 rpm for 10 min to separate the pelleted cells from the supernatant and collected in new tubes. The volumes of pelleted cells were also recorded and lysates made. Solid tumors in the abdominal walls and cavity were collected, weighed, and recorded. A portion of solid tumors was made cell lysate for Western blot analysis. The rest of the tumor was fixed in formaldehyde (Fisher), paraffinized, and sectioned for histology and immunohistochemistry staining.

### Western blot analysis

Lysates from cells grown in monolayers and from tumors were prepared as described [22–26]. Protein concentration of each lysate was determined and verified as described [22–26]. The expression of METCAM/MUC18 in the lysates from various cells lines/clones (5 μg proteins of each lysate) was determined by Western blot (WB) analysis [22–26] by using a chicken anti-human METCAM/MUC18 IgY as the primary antibody (1/300 dilutions) [22–26]. An AP-conjugated rabbit anti-chicken IGY (AP162A) from Chemicon (1/2000 dilutions) was used as the secondary antibody. Primary antibodies for detection of Bcl2 (N-19, SC-492), Bax (N-20, SC-493), and VEGF (A-20, SC-152) were rabbit polyclonal antibodies from Santa Cruz Biotech. The rabbit anti-human LDH-A polyclonal antibody was previously made in our group [33]. Those for detection of phospho-AKT (Ser473) (D9E, #4060), pan-AKT (C67E7, #4691), and VEGFR2 (53B11, #24790) were rabbit monoclonal antibodies from Cell Signaling Technology. The primary antibody for detection of PCNA (PC-10, SC-56, Santa Cruz Biotech) was a mouse monoclonal antibody. The 1/2000 dilution of the corresponding AP-conjugated secondary antibody, goat anti-rabbit antibody (AP132A), or rabbit anti-mouse antibody (AP160A) from Chemicon, was used. As the loading controls, the same WB membrane was reacted with three primary antibodies (1/200 dilutions) against three house-keeping genes, such as actin, β-tubulin, and GAPDH, which were goat polyclonal antibody (C-11, SC-1615), rabbit polyclonal antibody (H-235, SC-9104), and goat polyclonal antibody (SC-20358), respectively, from Santa Cruz Biotech. The 1/2000 dilution of AP-conjugated rabbit anti-goat (AP106A) or goat anti-rabbit (AP132A) antibody from Chemicon was used as the secondary antibodies. Substrates BCIP/NBT (S3771, Promega) were used for color development. The image of the specific protein band corresponding to METCAM/MUC18, each key downstream parameter, or each of the three house-keeping genes on the same membrane, was scanned by an Epson Scanner model 1260 and its intensity was quantitatively determined by a NIH software program Image J version 1.31.

### Histology and immunohistochemistry (IHC) of the tumor tissue sections

Paraffin-embedded tissue sections (5 μm) were de-paraffinized, rehydrated with graded alcohol and PBS, and used for histological staining (H&E) and IHC analyses [22–26]. A tissue section of *SC* tumors derived from the human prostate cancer LNCaP-expressing clone (LNS239) was used as a positive external control for IHC staining [22]. 1/200 to 1/300 dilution of the chicken anti-huMETCAM/MUC18 IGY antibody was used as the primary antibody and 1/250 dilution of the biotinylated rabbit anti-chicken IGY antibodies (G2891, Promega) as the secondary antibody [22–26]. A streptavidin-conjugated horseradish peroxidase complex (Dako LSAAB-2 system) and diaminobenzidine were used for color development. Hematoxylin was used as the counter staining. Negative controls had the primary antibody replaced by non-fat milk or control chicken IGY.

### Statistical analysis of data

All the data were statistically analyzed by the Student’s *t* test by using the 1 tailed distribution type1, 2, or 3 method. Two corresponding sets of data were considered significantly different if the *P* value was < 0.05.

## Results

### Expression of METCAM/MUC18 in various human ovarian cancer cell lines

**Fig. 1:**
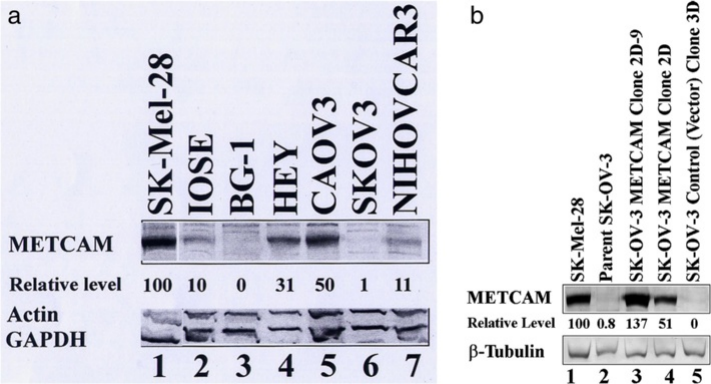
Expression of METCAM in various human ovarian cancer cell lines (**a**) and in G418^R^- clones derived from SK-OV-3 (**b**). **a** The expression of METCAM/MUC18 in the lysates from various cells lines was determined by Western blot (WB) analysis as described in “Methods”. Cell lysate from a human melanoma cell line, SK-Mel-28, was used as a positive control (lane 1) and those from human ovarian cancer cell lines, BG-1 (lane 3) and SK-OV-3 (lane 6) as negative controls. METCAM/MUC18 expression levels in cell lysates from one immortalized human ovarian epithelial cells (IOSE) and in five human ovarian cancer cell lines are shown in lanes 2 to 7. The number under each lane indicates the relative level of METCAM/MUC18 of each cell line, assuming that in SK-Mel-28 is 100 %. Only the house-keeping genes, actin and GAPDH, are shown here as the loading controls. **b** Human METCAM/MUC18 expression in lysates prepared from various clones/cells was determined by Western blot analysis as described in “Methods”. METCAM/MUC18 expression level in cell lysates from a human melanoma cell line, SK-Mel-28, was used as a positive control (lane 1) and from the parental human ovarian cancer cell line, SK-OV-3, as a negative control (lane 2). METCAM/MUC18 expression in cell lysates from one single SK-OV-3 clone (METCAM Clone 2D-9) and two pooled SK-OV-3 clones (METCAM Clone 2D and Control (Vector) Clone 3D) are shown in lanes 3–5. Both the METCAM Clone 2D-9 and the METCAM Clone 2D were derived from SK-OV-3 cells transfected with the human METCAM/MUC18 cDNA gene. The Control (Vector) Clone 3D was from SK-OV-3 cells transfected with the empty vector. The number under each lane indicates the relative level of METCAM/MUC18 of each cell line, assuming that in SK-Mel-28 was 100 %. β-tubulin is shown as the loading control

We initiated the investigation by determining expression levels of METCAM/MUC18 in several ovarian cancer cell lines. Figure 1a shows that the expression level of METCAM/MUC18 in one immortalized normal ovarian epithelial cell line (IOSE) was about 10 % and that in five ovarian cancer cell lines, BG-1, HEY, CAOV-3, SK-OV-3 and NIHOVCAR3, ranged from zero to 50 %, assuming that a positive control, human melanoma cell line SK-Mel-28, expressed 100 % of METCAM/MUC18. This provided an important information for us to choose two cell lines, BG-1 (established from a poorly differentiated adenocarcinoma) and SK-OV-3 (established from an adenocarcinoma metastasis as malignant ascites), which expressed very low levels of METCAM/MUC18 (zero and 1 %, respectively), for in vitro and in vivo studies. In this report, we have provided the results of the following studies by using the human ovarian cancer cell line, SK-OV-3. The results of similar studies by using the BG-1 cell line will be reported elsewhere.

### METCAM/MUC18 expression in G418^R^-clones derived from SK-OV-3 cells

Since the SK-OV-3 cell line does not express METCAM/MUC18, to determine if METCAM/MUC18 expression affects the in vitro and in vivo cellular behaviors of the cells, it would be desirable to ectopically make SK-OV-3 express the protein by transfecting the cells with the human METCAM/MUC18 cDNA. To facilitate the expression of the transfected gene, the cDNA is inserted in a mammalian expressible plasmid vector, pcDNA3.1+, in which the inserted gene is driven by a strong CMV promoter to facilitate the high expression of the inserted gene in mammalian cells. Since the pcDNA3.1+ also contains the cDNA encoding for neomycin (or G418)-resistant gene, which is driven by the SV40 promoter, the transfected cells should also express the neomycin-resistant gene and be resistant to the killing of neomycin (G418). As such, the majority of the cells, which were not successfully transfected with the plasmid, should be killed in the growth medium containing G418. In contrast, a minority of the cells, which were successfully transfected with the plasmid, should be resistant to the killing of G418 and enriched in the presence of G418; most of them should also express METCAM/MUC18, albeit at different levels in different clones. To obtain high expressing clones after transfecting SK-OV-3 cells with the human METCAM/MUC18 cDNA, the G418-resistant (G418^R^)-clones were selected and the expression level of METCAM/MUC18 in each clone was determined by Western blot analysis. The control cells, which were transfected with the empty vector that did not contain the human METCAM/MUC18 cDNA, should not express METCAM/MUC18 similar to the parental SK-OV-3 cells, even though they were G418^R^. We found that DEMRIE-C was an excellent transfecting reagent, since 2/3 were high-expressing clones. However, the transfecting reagent of FuGene HD (Roche) was not, since no high-expressing clones were obtained and 2/3 clones were low-expressing clones and 1/3 medium-expressing clones. Figure 1b shows that the expression of METCAM/MUC18 in three typical G418^R^ clones when DEMRIE-C was used as the transfecting reagent. When compared to the positive control cell line, human melanoma SK-Mel-28 cells (assuming expression of 100 % of METCAM/MUC18) (lane 1), the METCAM clone 2D-9 (lane 3) and the METCAM clone 2D (lane 4) of SKOV3 cells showed much higher expression of METCAM/MUC18 (137 and 51 %, respectively) than that of clone of the control (vector) clone 3D (lane 5), which expressed 0 % of METCAM/MUC18, similar to the parental SK-OV-3 cells (lane 2).

### Effects of METCAM/MUC18 expression on the cell motility and invasiveness in vitro

**Fig. 2:**
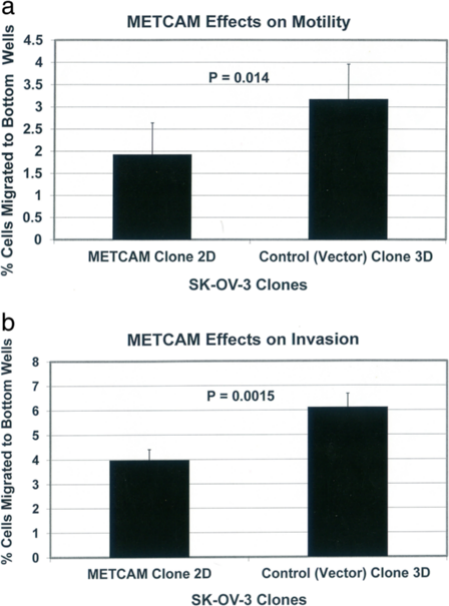
Effects of huMETCAM/MUC18 expression on the in vitro motility (**a**) and invasiveness (**b**) of SK-OV-3 clones/cells. **a** For the motility test, the METCAM clone 2D and the Control (Vector) clone 3D of SK-OV-3 cells were used. Six hours after seeding to the top wells, cells migrating to the bottom wells were determined as described in “Methods”. Means and standard deviations of triplicate values of the motility tests are indicated. *P* value, which was determined by analyzing two sets of data with the Student’s *t* test by using the one-tailed distribution-type 2 method, was 0.014, indicating that the result was statistically different. **b** For invasiveness test, the METCAM clone 2D and the Control (Vector) clone 3D of SK-OV-3 cells were used. Six hours after seeding cells to the top wells, cells migrating to the bottom wells were determined as described in “Methods”. Means and standard deviations of triplicate values of the invasiveness tests are indicated. *P* value, which was determined by analyzing two sets of data with the Student’s *t* test by using the one-tailed distribution-type 2 method, was 0.0015, indicating that the result was statistically different

Figure 2a shows the effect of METCAM/MUC18 over-expression on the motility of SK-OV-3 cells. As shown in Fig. 2a, the motility of the METCAM clone 2D, which expressed a high level of METCAM/MUC18, was 1.65-fold lower than that of the control (vector) clone 3D, which expressed 0 % of METCAM/MUC18. Figure 2b shows the effect of METCAM/MUC18 over-expression on the invasiveness of SK-OV-3 cells. As shown in Fig. 2b, the invasiveness of the METCAM clone 2D was 1.57-fold lower than that of the control (vector) clone 3D. Taken together, we conclude that increased METCAM/MUC18 expression decreased both motility and invasiveness of SK-OV-3 cells.

### METCAM/MUC18 expression inhibits in vivo tumorigenicity of SK-OV-3 cells in nude mice

**Fig. 3:**
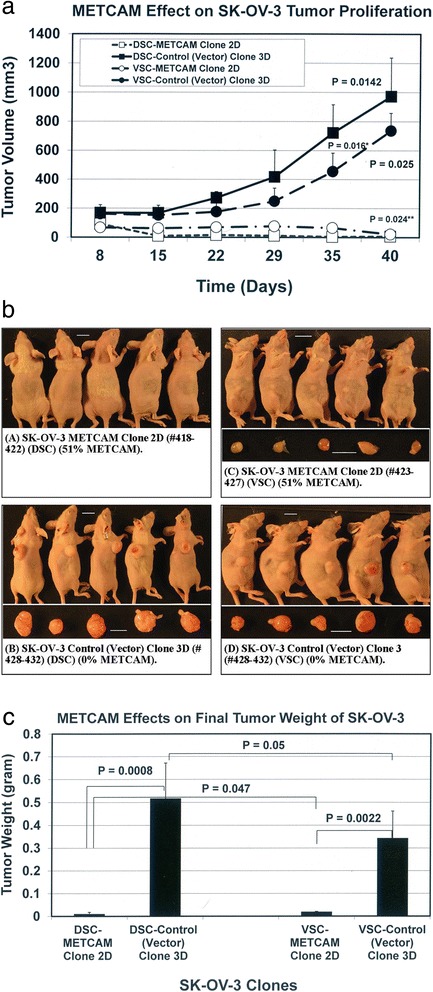
Effects of huMETCAM/MUC18 expression on the in vivo tumorigenesis of SK-OV-3 clones/cells at the *SC* injection sites. **a** Tumorigenicity of the METCAM clone 2D and the Control (Vector) clone 3D of SK-OV-3 was determined by subcutaneous injection of 5 × 10^6^ cells of cells from each clone at the dorsal and ventral sides in female athymic nude mice. Tumor proliferation by the two clones is shown by plotting mean tumor volumes/weights versus time after injection. *P* values were determined by analyzing all the data with the student’s *t* test by using 1-tailed distribution-type 1 method. *P* values between tumor volumes through the time course of the METCAM clone 2D and that of the control (vector) clone 3D were 0.0142 at the dorsal site and 0.025 for the ventral site of injection, respectively. *P* value between the dorsal and the ventral sites of the METCAM clone 2D was 0.024 (**) and that between the two sites of the control (vector) clone 3D was 0.016 (*). **b** The panels **a** and **b** show the mice bearing tumors from the METCAM clone 2D and the control (vector) clone 3D, respectively, at the dorsal sites (DSC). The panels **c** and **d** show the mice bearing tumors from the METCAM clone 2D and the control (vector) clone 3D, respectively, at the ventral sites (VSC). **c** The mean final tumor weights of the two clones injected at both dorsal and ventral sites in athymic nude mice were compared at the endpoint. Both the mean final tumor weights from five mice of the control (vector) clone 3D were statistically significantly heavier than the mean tumor weight from those of the METCAM clone 2D, since the *P* values, which were analyzed by the Student’s *t* test (one-tailed distribution-type 1 method) between the tumors from the METCAM clone 2D and the control (vector) clone 3D at the dorsal and ventral sites were 0.0008 and 0.0022, respectively. The *P* values of the final tumor weights analyzed by the Student’s *t* test (one-tailed distribution-type 1 method) between the dorsal and ventral sites were 0.047 for the METCAM clone 2D and 0.05 for the control (vector) clone 3D, respectively

The effect of METCAM/MUC18 over-expression on in vivo tumorigenicity of SKOV3 cells was determined in female nude mice after *SC* injection at either dorsal or ventral side. As shown in Figs. 3a and b, the tumor proliferation of the METCAM clone 2D was much lower than that of the control (vector) clone at both sites, indicating that over-expression of METCAM/MUC18 decreased tumorigenicity of SK-OV-3 cells in nude mice. Consistent with the results in Figs. 3a and b, Fig. 3c shows that final tumor weights of the METCAM clone 2D were also lower than those of the control (vector) clone 3D at both sites, indicating that over-expression of METCAM/MUC18 decreased the final tumor weights of SK-OV-3 cells in nude mice. Interestingly, as also shown in Fig. 3, tumorigenicity of the control clone 3D on the dorsal side was significantly better than that on the ventral side, in contrast tumorigenicity of the METCAM clone 2D on the ventral side was significantly better than that on the dorsal site. Taken together, we conclude that over-expression of METCAM/MUC18 suppressed in vivo tumorigenesis of SK-OV-3 cells in nude mice.

### Expression of METCAM/MUC18 in subcutaneous tumors derived from SK-OV-3 clones

**Fig. 4:**
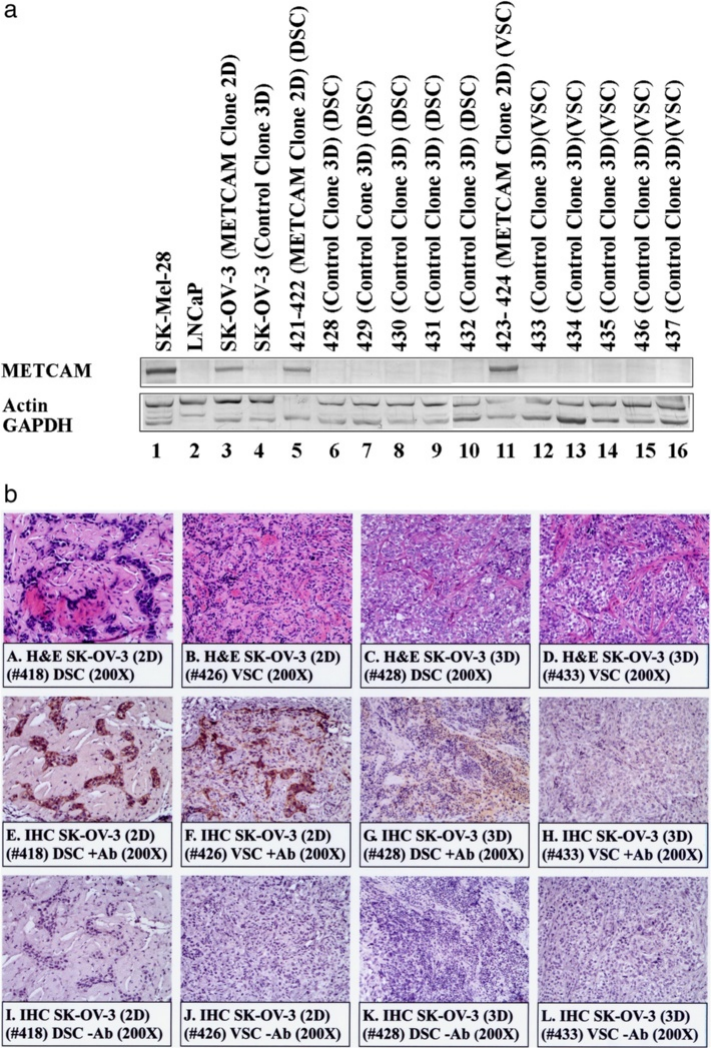
HuMETCAM/MUC18 expression in the subcutaneous tumors of SK-OV-3 clones/cells. **a** The expression of huMETCAM/MUC18 in the lysates from the tumors was determined by Western blot analysis as described in “Methods”. Lysates from SK-Mel-28 cells (lane 1) and from LNCaP cells (lane 2) were used as the positive control and the negative control, respectively. The huMETCAM/MUC18 expression levels in the tissue cultured cells of two SK-OV-3 clones, METCAM clone 2D and the control (vector) clones 3D, are shown (lanes 3–4) in comparison with those in the tumor lysates (lanes 5–16). The huMETCAM/MUC18 expression levels in the combined lysate from the two dorsal tumors (DSC) of the METCAM clone 2D (#421-422), the lysate of each of the five dorsal tumors (DSC) from the control (vector) clone 3D (#428-432), the combined lysate from two ventral tumors (VSC) from the METCAM clone 2D (#423-424), and the lysate of each of the five ventral tumors (VSC) from the control (vector) clone 3D (#433-437) are shown. As loading controls, the same membranes were reacted with antibodies against three house-keeping genes, but only actin and GAPDH are shown. **b** Histology and immunohistochemistry (IHC) of *SC* tumors of the two SK-OV-3 clones. Panels *a*–*d* show histology of the tumor sections from the two SK-OV-3 clones injected at dorsal (DSC) or ventral (VSC) subcutaneous sites. Panels *e* to *l* show the IHC of these tumor sections. A tissue section of *SC* tumors derived from the human prostate cancer LNCaP-expressing clone (LNS239) was used as a positive external control for IHC staining (data not shown). IHC of all tumor sections were carried out as previously described in “Methods”. Panels *e* to *h* show the anti-huMETCAM/MUC18 antibody staining of the cells in the tumor sections. Both tumor sections (DSC and VSC) from the METCAM clone 2D showed strong brown color staining in IHC when the antibody was added (Panels *e & f*), however, the two tumor sections (DSC and VSC) from the control (vector) clone 3D showed a weak background staining (Panels *g & h*). Panels *i* to *l* show the corresponding negative controls which show no staining in the adjacent sections when no antibody or when the control chicken IgY was added

Figure 4a shows results of Western blot analysis that METCAM/MUC18 was not expressed in tumors derived from the control (vector) clone 3D, but was expressed in tumors derived from the METCAM clone 2D. Since the apparent electrophoretic mobility of the proteins from tumors in the gel (lanes 5–16) was similar to that from the tissue culture cells before injection (lanes 3–4), we concluded that the tumors were from the injected clones/cells. The IHC results in Fig. 4b showed that the tumor sections from the METCAM clone 2D (panels e and f) were stained much stronger than those from the control (vector) clone 3D (panels g and h), consistent with the Western blot results in Fig. 4a.

It is intriguing to find that the tumors derived from the METCAM clone 2D were barely visible with the naked eye, but visible under microscope in the tumor sections (Fig. b, panels a and b in H&E stain and panels e and f in IHC), which appeared to be confined to small regions, whereas tumors derived from the control (vector) 3D were not confined (Fig. 4b, panels c and d in H&E stain and panels g and h in IHC).

### METCAM/MUC18 expression inhibits tumorigenicity and ascites formation of SK-OV-3 cells in the abdominal cavity of nude mice

**Fig. 5:**
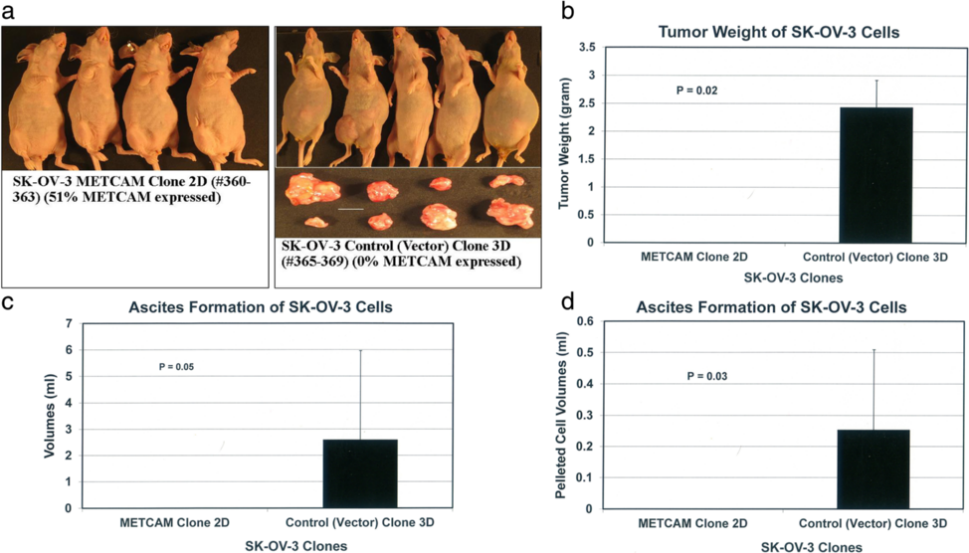
Effect of huMETCAM/MUC18 expression on the in vivo tumorigenesis of SK-OV-3 clones/cells at the *IP* injection sites. Tumorigenicity of the METCAM clone 2D and the control (vector) clone 3D of SK-OV-3 was also determined by *IP* injection of 5 × 10^6^ cells of the two clones in athymic nude mice. **a** Shows that the mice injected with the control (vector) clone 3D bore tumors and developed ascites in the intra-peritoneal cavity (#365-369), whereas the mice injected with the METCAM clone 2D did not bear any tumors and develop ascites in the intra-peritoneal cavity (#360-363). The bar shows the 1 cm mark. **b** Shows the final mean tumor weights of the two clones in the abdominal cavity, and **c** the final volumes of total ascites fluid and **d** the pelleted volume of ascites cells of both clones. *P* values were determined by analyzing all the data with the Student’s *t* test by using the 2-tailed distribution-type 1 method in (**b**) and the 1-tailed distribution-type 1 method in (**c**) and (**d**)

To further determine the effect of METCAM/MUC18 over-expression on in vivo tumorigenicity of SK-OV-3 cells in the orthotopic site (*IP* cavity), SK-OV-3 cells from the METCAM clone 2D and the control (vector) 3D were *IP* injected into female nude mice. As shown in Fig. 5a, the mice in the control group, which were injected with the control (vector) clone 3D, developed swollen abdominal cavity, but not the mice in the test group, which were injected with the METCAM clone 2D. After dissection of the abdominal cavities, we found that tumors and ascites were formed in four of five mice in the control group, whereas no tumors and ascites were found in the test group (Figs. 5b–d). Consistent with the observation, the final weights of abdominal tumors and volumes of ascites were measured, and were significantly heavier in the group injected with the control (vector) clone 3D than those injected with the METCAM clone 2D, as shown in Figs. 5b–d. We concluded that over-expression of METCAM/MUC18 suppressed the tumorigenicity and ascites formation of SK-OV-3 cells in *IP* cavities in nude mice.

### Expression of METCAM/MUC18 in abdominal tumors and ascites derived from SK-OV-3 clones

The METCAM/MUC18 expression in the *IP* tumors and ascites formed by the vector control 3D clone in mice was also determined by Western blot analysis. The results showed that METCAM/MUC18 was minimally detectable in the ascites and tumors similar to the parental SK-OV-3 cells (data not shown), suggesting that those tumors were from the injected SK-OV-3 clones.

### Preliminary mechanisms of METCAM/MUC18-mediated suppression of the progression of SK-OV-3 cells

**Fig. 6:**
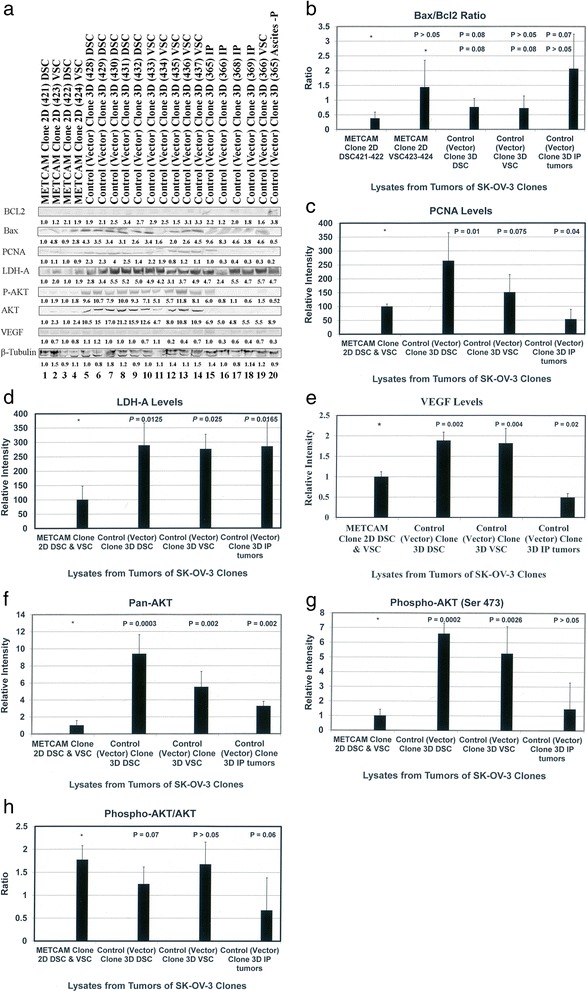
The effect of METCAM/MUC18 expression on levels of various key parameters expressed in the tumor lysates, which may affect the tumor growth**.** Tumor lysates were used in the western blot analysis by using various antibodies, as described in “Methods”. **a** The summary of Western blot results of levels of various key parameters are shown, and their quantitative results are shown in (**b**) Bax/BCl2 ratios, **c** PCNA, **d** LDH-A, **e** VEGF, **f** pan-AKT, **g** Phospho-AKT(Ser473), and **h** Phospho-AKT(Ser473)/pan-AKT ratios. *P* values were determined by using the Student’s *t* test (1-tailed distribution-type 2/3 method) to analyze the data between the tumor lysates from the METCAM clone 2D clone and those from the Control (Vector) Clone 3D

Mechanisms of METCAM/MUC18-mediated suppression of the progression of human ovarian cancer cells have not been studied. By deducing knowledge learned from METCAM/MUC18-induced tumorigenesis of other tumor cell lines, such as, melanoma, cancers in breast and prostate and nasopharyngeal carcinoma, METCAM/MUC18 may affect tumorigenesis by cross-talk with many downstream signaling pathways that regulate proliferation, survival pathway, apoptosis, metabolism, and angiogenesis of tumor cells [7, 22–25]. To investigate if METCAM/MUC18-mediated tumor suppression also affected expression of its downstream effectors, such as indexes of apoptosis/anti-apoptosis, proliferation, survival, aerobic glycolysis, and angiogenesis, we determined the expression of levels of Bcl2, Bax, PCNA, LDH-A, VEGF, pan-AKT, phospho-AKT(Ser 473), and the ratio of phospho-AKT/AKT in tumor lysates. Figure 6a shows the Western blot results of the expression levels of Bcl2, Bax, PCNA, LDH-A, VEGF, pan-AKT, and phospho-AKT (Ser473) in tumor lysates. Figure 6b shows that the ratios of Bax/Bcl2 were not statistically different between tumors derived from the METCAM clone 2D and those from the control (vector) clone 3D, indicating that over-expression of METCAM/MUC18 did not affect apoptosis or anti-apoptosis of SK-OV-3 cancer cells during in vivo tumorigenesis. Figures 6a and c show that tumor lysates from the METCAM clone 2D had a lower level of PCNA than the control (vector) clone 3D, indicating that over-expression of METCAM/MUC18 decreased proliferation of SK-OV-3 cancer cells during in vivo tumorigenesis. Figures 6a and d show that tumor lysates from the METCAM clone 2D had a lower level of LDH-A than the control (vector) clone 3D, indicating that over-expression of METCAM/MUC18 decreased proliferation of SK-OV-3 cancer cells by decreasing aerobic glycolysis during in vivo tumorigenesis. Figures 6a and e show that tumor lysates from the METCAM clone 2D had a lower level of VEGF than the control (vector) clone 3D, indicating that over-expression of METCAM/MUC18 decreased proliferation of SK-OV-3 cancer cells by decreasing angiogenesis during in vivo tumorigenesis. Figures 6a and f show that the level of pan-AKT was lower in tumors from the METCAM clone 2D than those from the control (vector) clone 3D, indicating that over expression of METCAM/MUC18 decreased the expression of pan-AKT. Figures 6a and g show that phospho-AKT (Ser473) was lower in tumors from the METCAM clone 2D than those from the control (vector) clone 3D, indicating that over expression of METCAM/MUC18 decreased the expression of phospho-AKT (Ser473), which in turn affects motility and cell growth. Figures 6a and h show that ratios of phospho-AKT (Ser 473)/AKT in tumors of the METCAM clone 2D was not statistically significantly different from those in tumors of the control (vector) clone 3D, indicating that METCAM over-expression did not affect the survival pathway of SK-OV-3 cancer cells during in vivo tumorigenesis. Taken together, we suggest that over expression of METCAM/MUC18 may suppress tumorigenesis and malignant progression of ovarian cancer cells in nude mice by decreasing their abilities in proliferation, aerobic glycolysis, and angiogenesis, and by decreasing motility and invasiveness, but not altering the apoptosis/anti-apoptosis and survival pathways.

## Discussion

In this study, we initiated the investigation by determining expression levels of METCAM/MUC18 in several ovarian cancer cell lines. We found that METCAM/MUC18 was expressed at a level of 31–50 % in two out of three cell lines established from primary adenocarcinomas (HEY and CAOV3), but poorly expressed (1–11 %) in two cell lines established from malignant ascites (SKOV3 and NIHOVCAR3). It appeared that METCAM/MUC18 was expressed poorer in malignant cell lines than in primary adenocarcinomas, suggesting that METCAM/MUC18 may play a negative role in the progression of ovarian cancer. To further support this hypothesis, we provided in vitro evidence to show that a high expression level of METCAM/MUC18 inhibited the migration and invasion of SKOV3 cancer cells. We also provided in vivo evidence in animal tests to show that METCAM/MUC18 expression inhibited the tumorigenicity at the subcutaneous sites as well as the tumorigenicity and ascites formation in the intra-peritoneal cavity of an athymic nude mouse model. Since the METCAM/MUC18 expressed in the tumors and ascites cells were similar to that in the injected clones/cells, the protein was not modified to manifest these processes. Taken together, we conclude that METCAM/MUC18 serves as a tumor suppressor as well as a metastasis suppressor for the human ovarian cancer cells SK-OV-3. METCAM/MUC18 may suppress tumorigenesis and malignant progression of ovarian cancer cells in nude mice by decreasing their abilities in proliferation, aerobic glycolysis, and angiogenesis, and by decreasing their abilities in EMT, but not altering the apoptosis/anti-apoptosis and survival pathways.

This conclusion contradicts the results of a positive correlation of clinical prognosis with the increased expression of METCAM/MUC18 in malignant ovarian cancer specimens [17, 18, 29]. This suggests that the positive correlation in this case is fortuitous and that we should not assume a positive role of METCAM/MUC18 in the progression of ovarian cancer without the support of tests in an animal model. Our results also contradict the previously established notion that METCAM/MUC18 serves as a tumor promoter in both prostate cancer cells [22] and breast cancer cells [23, 24], and as a metastasis promoter in human melanoma cells [25], prostate cancer [26], and breast cancer [27]. The conclusion, nevertheless, appears to be consistent with the first notion suggested by one group that METCAM/MUC18 is a tumor suppressor in human breast cancer cell line MCF-7 [28]; albeit the notion was later proven to contradict to the evidence from two different groups [23, 24, 27]. Regardless, the role of METCAM/MUC18 as a tumor suppressor was not only conclusively demonstrated in a human ovarian cancer cell line, SK-OV-3 (as shown here), but also in another human ovarian cancer cell line BG-1 [Wu, unpublished results], as well as in a mouse melanoma cell line, K1735-9 [34] and one NPC cell line, NPC-TW01 ([35, 36], & Wu, unpublished results). METCAM/MUC18 has also been demonstrated as a metastasis suppressor in the two human ovarian cancer cell lines, SK-OV-3 (as also shown here) and BG-1 [Wu, unpublished results], and one mouse melanoma cell line, K1735-9 [34]. Thus sufficient evidence is provided to support the novel suppressor role of METCAM/MCU18 in the progression of these human cancers.

E-cadherin, a cell adhesion molecule, has been demonstrated as a tumor suppressor role in many tumors derived from epithelium; however, E-cadherin has not been found to play a tumor or metastasis promoter role in any tumor [8]. Thus the most intriguing, unique biological function of METCAM/MUC18 in tumorigenesis and metastasis is that it seems to play a dual role in the progression of some tumor cell lines. It can be a tumor/metastasis promoter in prostate cancer cell lines [22, 26], breast cancer cell lines [23, 24, 27], and most melanoma cell lines [19, 25, 34]. It can also be a tumor/metastasis suppressor in the progression of other tumor cell lines in animal studies, such as, two ovarian cancer cell lines (in this report and Wu, unpublished results), one mouse melanoma subline ([34] and Wu, unpublished results), nasopharyngeal carcinoma ([35, 36] and Wu, unpublished results), and perhaps hemangioma [37]. It is not clear why METCAM/MUC18 plays a dual role in tumorigenicity and metastasis. One point is clear, which is that METCAM/MUC18 plays an opposite role in different cancer types or in different clones/sublines of the same cancer type [38]. Thus it is logical to propose that the effect of METCAM/MUC18 on the progression of epithelial cancers is modulated by different intrinsic factors in different tumor cells/types. The dual role of METCAM/MUC18 is very likely due to the presence of different interacting partners intrinsic to each cancer cell type and different clone, or perhaps due to different heterophilic ligands, which unfortunately have not been identified [19, 34, 38]. Interactions of METCAM/MUC18 with different sets of intrinsic partners may result in the promotion or suppression of tumorigenicity and metastasis via increasing or decreasing aerobic glycolysis, proliferation, angiogenesis, other growth-promoting pathways, as well as altering tumor cell motility, invasiveness, and vascular metastasis, as suggested in this report. In the future, the identification of these partners and/or ligands is essential to understand further detailed mechanisms.

Interestingly, many molecules have recently been shown to play a dual role in the progression of cancer. The most well-known examples are TGF-β, which is context dependent and acts as a tumor suppressor in the early stage of tumorigenesis, but as a progression promoter in the late stage [7], and VEGF, which also plays a dual role in tumor progression [39].

One point worth noting is that the tumors induced by the METCAM clone 2D were confined to small regions, as shown in the results of H&E and IHC, whereas the tumors induced by the control (vector) clone 3D developed serious tumors, suggesting that tumors from the 2D clone appeared to be dormant; thus METCAM/MUC18 may function similarly to other tumor suppressors in other tumor cells [40].

Another point also worth noting is that tumorigenicity of the control (vector) clone 3D in the dorsal site appeared to be significantly better than that in the ventral site (*P* value = 0.016), whereas tumorigenicity of the 2D clone in the ventral site was significantly better than that in the dorsal site (*P* value = 0.024). We don’t know why different *SC* sites have different effects on tumorigenicity. This also requires further investigation.

## Conclusion

In summary, we have provided the first conclusive evidence to suggest that human METCAM/MUC18 is a novel suppressor in the progression of human ovarian cancer. The notion is supported by the evidence that the over-expression of human METCAM/MUC18 inhibited in vitro motility and invasion and in vivo tumor formation of a human ovarian cancer cell line, SK-OV-3, at *SC* sites as well as in the *IP* cavities of an athymic nude mouse model. It also inhibited in vivo ascites formation of SKOV3 cells in the mouse *IP* cavities. The tumor/metastasis suppressor role of human METCAM/MUC18 in the progression of human ovarian cancer cells is opposite to its role in breast cancer, prostate cancer, and most melanoma cell lines. This novel role of METCAM/MUC18 is not unique in this human ovarian cancer cell line, but is also found in another human ovarian cancer cell line, BG-1, one mouse melanoma subline, and one nasopharyngeal cancer cell line. The dual role played by METCAM/MUC18 in the progression of different cancers may be dependent upon the unique intrinsic constituents and cell surface heterophilic ligands in different cancer cell types, which require future investigation. How METCAM/MUC18 affects tumor dormancy should also be an interesting aspect for future investigation, since tumor dormancy may be due to intrinsic growth inhibition, immunological suppression, and/or angiogenic suppression [40].

## Abbreviations

CAM: cell adhesion molecule
FBS: fetal bovine serum
G418^R^: G418-resistant
huMETCAM/MUC18: human METCAM/MUC18
IHC: immunohistochemistry
METCAM: metastasis cell adhesion molecule
*IP*: intraperitoneal
*SC*: subcutaneous
